# Investigation of Channel Selection for Gesture Classification for Prosthesis Control Using Force Myography: A Case Study

**DOI:** 10.3389/fbioe.2019.00331

**Published:** 2019-12-10

**Authors:** Chakaveh Ahmadizadeh, Brittany Pousett, Carlo Menon

**Affiliations:** ^1^Menrva Research Group, Schools of Mechatronic Systems Engineering and Engineering Science, Simon Fraser University, Metro Vancouver, BC, Canada; ^2^Barber Prosthetics Clinic, Vancouver, BC, Canada

**Keywords:** force myography, gesture classification, channel selection, prosthesis control, robotic hand, high density FMG, upper limb prosthesis

## Abstract

**Background:** Various human machine interfaces (HMIs) are used to control prostheses, such as robotic hands. One of the promising HMIs is Force Myography (FMG). Previous research has shown the potential for the use of high density FMG (HD-FMG) that can lead to higher accuracy of prosthesis control.

**Motivation:** The more sensors used in an FMG controlled system, the more complicated and costlier the system becomes. This study proposes a design method that can produce powered prostheses with performance comparable to that of HD-FMG controlled systems using a fewer number of sensors. An HD-FMG apparatus would be used to collect information from the user only in the design phase. Channel selection would then be applied to the collected data to determine the number and location of sensors that are vital to performance of the device. This study assessed the use of multiple channel selection (CS) methods for this purpose.

**Methods:** In this case study, three datasets were used. These datasets were collected from force sensitive resistors embedded in the inner socket of a subject with transradial amputation. Sensor data were collected as the subject carried out five repetitions of six gestures. Collected data were then used to asses five CS methods: Sequential forward selection (SFS) with two different stopping criteria, minimum redundancy-maximum relevance (mRMR), genetic algorithm (GA), and Boruta.

**Results:** Three out of the five methods (mRMR, GA, and Boruta) were able to decrease channel numbers significantly while maintaining classification accuracy in all datasets. Neither of them outperformed the other two in all datasets. However, GA resulted in the smallest channel subset in all three of the datasets. The three selected methods were also compared in terms of stability [i.e., consistency of the channel subset chosen by the method as new training data were introduced or some training data were removed (Chandrashekar and Sahin, 2014)]. Boruta and mRMR resulted in less instability compared to GA when applied to the datasets of this study.

**Conclusion:** This study shows feasibility of using the proposed design method that can produce prosthetic systems that are simpler than HD-FMG systems but have performance comparable to theirs.

## 1. Introduction

This section provides an application background, explains the motivation of this study, and outlines some of the related work. It also provides an introduction to channel selection and its various categories.

### 1.1. Application Background

The quality of life of an individual with upper limb amputation could be drastically affected by their decreased autonomy as a result of the loss of their limb (Castellini and Koiva, 2013). Powered prostheses can provide users with means to gain back some of their upper limb functionality and subsequently increase their self-sufficiency. Reliability and intuitiveness of prostheses control play important roles in user experience and are amongst factors lack of which can lead to prostheses abandonment (Biddiss and Chau, 2007; Ahmadizadeh et al., 2017). Issues concerning these factors are commonly addressed through advancements in robustness of human machine interfaces (HMIs) used for prostheses control and through the use of pattern recognition for more intuitive control.

Various invasive and non-invasive HMIs have been introduced for the control of upper limb prostheses. Some of the non-invasive HMIs for this application that have gained interest in the research community are: gaze tracking (Castellini and Koiva, 2013), electromyography (EMG) (Castellini et al., 2009; Scheme and Englehart, 2011), electroneurography (ENG) (Cloutier and Yang, 2013), mechanomyography (MMG) (Xiloyannis et al., 2015), force myography (FMG) (Rasouli et al., 2015), etc.

Recent studies have demonstrated FMG to be a promising HMI for upper limb prosthesis control (Cho et al., 2016; Radmand et al., 2016; Ahmadizadeh et al., 2017; Jiang et al., 2017; Sadeghi Chegani and Menon, 2018). FMG monitors changes in volumetric pattern of user's forearm and detects intentions of the user based on these changes (Rasouli et al., 2016). Various methods are used to monitor these volumetric variations, one the most common of which is the use of force sensitive resistors (FSRs) (Castellini and Ravindra, 2014; Jiang et al., 2017).

A study by Jiang et al. showed that FMG has the potential to outperform the more traditionally accepted HMI, i.e., sEMG, for hand gesture classification (Jiang et al., 2017). In this study, Jiang et al. reported classification accuracies of as high as 83.5% for 48 static hand gestures using 8 FSRs with 12 healthy participants. Ferigo et al. reported classification accuracies of 81.2 and 72.8% for 6 and 11 static gestures, respectively, in a case study with one participant with transradial amputation (Ferigo et al., 2017). Cho et al. reported 62.61 and 41.73% classification accuracies for 6 and 11 static hand gestures, respectively, by placing eight FSRs on the residual limbs of 4 subjects with transradial amputations (Cho et al., 2016).

High density FMG is a potentially enhanced alternative for low density FMG. Belyea et al. conducted a study to demonstrate possibility of using high density force myography (by using a grid of 16 by 24 pressure sensors) for proportional control of upper limb prostheses (Belyea et al., 2018). Radmand et al. also investigated high-density force myography for upper limb powered prosthesis control (Radmand et al., 2016). Despite the potential increased performance of high density sensory systems, their higher cost in terms of computation, signal acquisition hardware, and problem complexity can lead to their incompatibility for many applications. Reduction of complexity of such systems could be possible through the use of channel selection.

Channel selection (CS) is a technique that reduces the dimension of input data by removing irrelevant input variables while maintaining the ones with vital information in the selected feature subset. The final channel subset leads to no or little reduction of performance of the system (Thiemjarus et al., 2004; Yu et al., 2006). In cases without feature extraction, such as this study, input channels (sensor data in this study) and input features are the same (Wang et al., 2017), and channel selection is the same as feature selection. Moving forward, in this article, the term “feature selection (FS)” is used due to its more common use in this field and others.

Feature selection methods mostly involve iterations of two steps until their stopping criterion is met (Deng et al., 2008). The two iterative steps are feature subset selection and evaluation of performance of the chosen subset (Deng et al., 2008). FS algorithms utilize various search techniques for selection of feature subsets (Deng et al., 2008) based on which they fall under three main categories: filter methods, wrapper methods, and embedded methods (Pal and Foody, 2010; Xu et al., 2010; Yan and Zhang, 2015).

Filter methods generally rank features based on a scoring criterion with no dependence on any classifier (Yan and Zhang, 2015). Wrapper methods select feature subsets based on their classification performance. These methods directly utilize the corresponding classifier as a wrapper for their search mechanism (Ding and Peng, 2005; Xu et al., 2010; Duro et al., 2012). Based on the search strategy used for feature subset selection, wrapper methods are divided into two main categories: sequential selection algorithms and heuristic search algorithms (Chandrashekar and Sahin, 2014). Embedded methods incorporate feature selection into the classification training process to reduce the cost of re-classification in wrapper methods (Deng et al., 2008; Chandrashekar and Sahin, 2014).

The use of feature selection in gesture classification has been explored in multiple studies. Wang et al. conducted a study to investigate feasibility of using FS for channel optimization of sEMG for gesture recognition. In this study, they used Genetic Algorithm to select channels based on data recorded from six able-bodied participants performing 13 gestures. They reported classification accuracy of 72.3% (97% of maximum accuracy that is obtained using all channels) after 0.125% reduction in the number of channels (Wang, 2019). Li et al. also conducted a study in which they performed channel selection for both sEMG and EEG that were used in fusion for control of upper limb prostheses. This study investigated data from four individuals with amputations performing five motion classes. In this study, Sequential Forward Selection was used for channel selection from 32 sEMG and 64 EEG channels. They reported classification accuracies of 84.2 and 87.0% for two optimized channel numbers (10 sEMG and 10 EEG channels, 10 sEMG and 20 EEG channels). The maximum classification accuracy (91.7%) was obtained when all channels were used (Li et al., 2017).

Dimension reduction for FMG HMI systems has been attempted by Radmand et al. (2016) and Jiang et al. (2017). Jiang et al. utilized a sequential forward feature selection algorithm for selection of 8 sensors out of a total of 16 sensors (Jiang et al., 2017). They reported a statistically significant decrease in accuracy of the system due to this reduction in the number of sensors (Jiang et al., 2017). Radmand et al. conducted a study that used channel reduction to reduce input dimension. In this study, reduction of groups of channels was investigated. Channels were grouped based on location of their corresponding sensor in the matrix. They reported that they were able to maintain classification accuracy with a lower density of sensors with some of the channel reduction options they explored (Radmand et al., 2016). To the best of our knowledge, no study has attempted feature selection for high density FMG without grouping sensors based on their location.

In addition to robustness of the HMI used for prostheses control, intuitiveness of control also affects prostheses user satisfaction. Pattern recognition plays an important role in enhancement of this aspect of user experience in prostheses applications. Various classification methods have been assessed for FMG controlled prostheses, some of the commonly used ones of which are linear discriminant analysis (LDA), support vector machine (SVM), and k-nearest neighbors (KNN) (Naik et al., 2015; Ahmadizadeh et al., 2017). Amongst these, LDA is one of the most widely used classifiers due to its capability in separating different classes of gestures and also its computational efficiency (Cho et al., 2016; Radmand et al., 2016; Xiao and Menon, 2017a). A study by Ahmadizadeh et al. compared performance of the three aforementioned classifiers for gesture classification using FMG data and determined LDA to be the classifier of choice for their study (Ahmadizadeh et al., 2017).

Another classification method that has recently gained attention in research communities for gesture recognition is deep learning. Through the availability of technologies that allow for collection and accessibility of large datasets used for gesture recognition and activity tracking (e.g., smart watches, Microsoft's Kinect, and data management and sharing systems), deep learning has become a promising method for such applications (Phinyomark and Scheme, 2018). Various modalities are used as inputs for these deep learning models including RGB images, skeletal data, depth information, audio, video, etc. (Asadi-Aghbolaghi et al., 2017). Huang et al. used a novel 3D convolutional Neural Network (CNN) for sign language recognition which implicitly extracts features from the input video stream (Huang et al., 2015). Similarly, Molchanov et al. employed 3D CNN for hand gesture recognition for touchless control in automotive applications (Molchanov et al., 2015).

Recent expansion of EMG data sources due to availability of benchmark datasets and also development of high-density EMG systems providing spatial and temporal information about muscle activities, has made deep learning approaches relevant for EMG data and thus a candidate for prosthetic control applications (Phinyomark and Scheme, 2018). Georgi et al. used Hidden Markov Models (HMMs) for gesture recognition using an Inertial Measurement Unit (IMU) and HD-sEMG data from five subjects and 12 gestures and reported recognition rates of about 97.8 and 74.3% for session-independent and for person-independent cases, respectively (Georgi et al., 2015). In another study, Du et al. used deep convolutional networks for HD-sEMG data from 23 participants performing up to 12 gestures for inter-session gesture recognition in muscle computer interface applications. They reported recognition accuracies of 63.3 and 55.3% for 12 basic finger movements in inter-session and inter-subject cases, respectively (Du et al., 2017). Geng at al. also conducted a study employing deep convolutional networks for sEMG data for gesture recognition application. They reported within-subject recognition accuracy of 89.3% for 8 gestures using an instantaneous frame of sEMG image (Geng et al., 2016).

In activity/gesture recognition tasks like the one studied in this article, when practical use case of the application including dynamic movements of the body is considered, temporal dependencies are introduced (Asadi-Aghbolaghi et al., 2017). For consideration of such dependencies, Long Short-term Memories (LSTMs) have gained an important role in activity recognition using sequential input data. LSTMs improve on the drawback of the more traditionally used Recurrent Neural Networks (RNNs) which is their short-term memory (Asadi-Aghbolaghi et al., 2017). A study by Donanue et al. showed that considering time domain in addition to visual domain can improve upon methods that focus only on visual domain or methods that consider an instance of the visual representation of the input data. This study explored the use of Long-term Recurrent Convolutional Network (LRCN) for a variety of vision tasks including activity recognition based on video input for this comparison (Donahue et al., 2017). Other studies have also employed long short-term memory (LSTM) for gesture recognition for its capability to learn activities of varying time length (Nishida and Nakayama, 2016; Tsironi et al., 2016).

A study by Tsironi et al. explored the combination of CNN and LSTM (CNNLSTM) for gesture recognition. This combination is used due to its ability to implicitly extract features and handling variable-length sequences of data representing an activity/gesture. They employed CNNLSTM for recognition of 9 gesture classes for human robot interaction application. They analyzed data collected from an RGB camera from six subjects and obtained precision percentage of 73.33–100% for various gestures (Tsironi et al., 2017). To the best of authors' knowledge no study has used deep learning methods for FMG data.

FMG has been processed with various classification methods in various studies, but in most cases, the difference between them was not statistically significant (Cho et al., 2016; Radmand et al., 2016; Ahmadizadeh et al., 2017; Xiao and Menon, 2017a,b). Whereas, the influence of feature selection, which is the focus of this study, is critical in this experiment. As a result, for all classification purposes LDA was used in this study due to its good performance for baseline accuracy (which is the LOOCV accuracy using all features), and its simplicity. It is one of the limitations of this study that it does not consider deep learning methods. However, due to the specific application considered here (i.e., use of feature selection in the design phase for custom prostheses), size of available data is limited since it is specific to the individual and is collected only in the design phase. This prevents the use of deep learning in this application. In future work, it is valuable to consider deep learning methods with data collected from multiple subjects as sensor placements are fixed in all cases. With availability of larger datasets, it would be valuable to also explore application of LSTM for gesture detection. This can be especially valuable in the practical use case of prostheses control since a gesture is combined with dynamic movements of the arm which introduces temporal dependencies of input sequential data.

This study explores the use of feature selection for dimension reduction in a high density FMG system without grouping sensors. This is done toward the goal of achieving a design method that uses feature selection for FMG HMI to achieve performance of high density FMG in systems with lower cost and complexity. This could be attained by collecting data from the user with a high density system, determining location of features with the most contribution to gesture recognition, and finally designing a custom device for the individual that embeds sensors in selected locations. To examine the feasibility of this method, feature selection methods were assessed for reduction of input dimension in this case study.

Three datasets were used in this study. All three datasets were collected from the same individual with a transradial amputation using the same custom-made socket with FMG sensors embedded inside to control an off-the-shelf prosthetic hand. All datasets were collected using protocols including the same six gestures essential to activities of daily living. One of the datasets was collected using a static protocol and the other two with a dynamic protocol. Different sensor configurations were used in these datasets. The three datasets were used to include data from various sensor configurations, sample sizes and with both static and dynamic protocols. The analysis performed on all datasets was the same. This is a case study based on one specific individual and a specific prosthetic device used. For this experiment, a custom-made prosthetic socket was used to be compatible with the off-the-shelf prosthetic hand. For this reason and also due to the difficulty of recruiting subjects with amputation, all datasets were collected from one individual. This is one of the limitations of this preliminary study. In future work, more individuals should be considered.

This study explored the use of four feature selection algorithms for the three collected datasets. These algorithms contain one commonly used method from each category of FS algorithms: sequential forward selection (SFS), minimum redundancy, maximum relevance (mRMR), Genetic algorithm (GA), and Boruta [an embedded method that uses Random Forest (RF)]. These are explained in more detail in the “Materials and Methods” section.

Findings of this study indicated that mRMR, GA, and Boruta were able to decrease the number of input features considerably without significantly decreasing classification accuracy. This shows the feasibility of the proposed method to use HD-FMG in the design phase to reduce complexity of custom FMG controlled prostheses without compromising the performance of the system.

## 2. Materials and Methods

**Figure 1:**
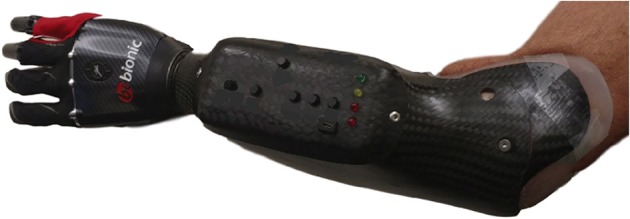
Custom made prosthetic socket and the Bebionic 3 robotic hand.

This section explains the materials and methods that were used to conduct data collection and data analysis for this study. A high density FMG controlled powered upper limb prosthesis was custom designed for the pilot subject using an off-the-shelf robotic hand as shown in Figure 1 to collect data in protocols consisting of hand gestures essential to activities of daily living. Collected data were then analyzed to determine which sensors had the most impact on the accuracy of classification of intended gestures.

Experiments of this study were approved by the Office of Research Ethics at Simon Fraser University and the participant provided informed consent.

### 2.1. Subject

In this study, data were collected from a right handed, 59 years old male with a transradial amputation of the left arm acquired in a work-related accident in 1980. He had experience using an EMG controlled powered prosthesis for 2 years, but used a body powered mechanical hook prosthesis on a daily basis. The subject was recruited by Barber Prosthetics Clinic (BPC).

### 2.2. Data Collection

#### 2.2.1. Hardware

**Figure 2:**
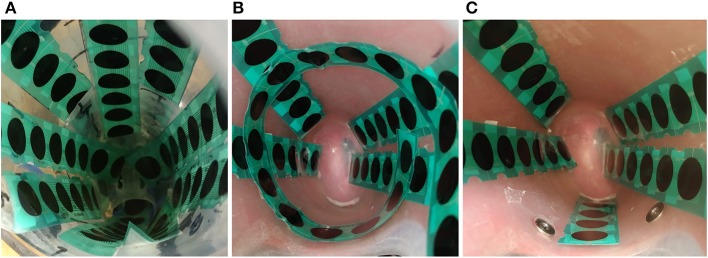
FSR strips placed inside the inner socket representing: **(A)** the sensor configuration for dataset1, **(B)** the sensor configuration used for dataset2, and **(C)** the sensor configuration used for dataset3.

Data were collected from the pilot subject wearing a custom-made prosthetic socket and a robotic hand (medium Bebionic 3, Ottobock, Duderstadt, Germany). The inner surface of the inner socket was covered with custom printed FSR strips each containing 16 0.5-in sensors similar to the FSR 402 from Interlink Electronics (Camarilo, California) (Ahmadizadeh et al., 2017). Three datasets were collected with different sensor placements and with both dynamic and static protocols to reduce bias of analysis based on a specific sensor placement or protocol. The number of strips and their configuration were different for the three different datasets. In dataset1, nine FSR strips were located in the socket so that it was covered by sensors as much as the physical shape of the socket allowed as shown in Figure 2. Not all sensors on the strips were located inside the socket. In the configuration used for dataset1, 63 sensors were located inside the socket to cover the inner surface of the socket as much as its physical shape allowed as shown in Figure 2. For dataset2, four strips were located on the extensor and flexor muscles to cover the muscle bellies. Another strip was located around the forearm over the muscle bellies in a circumferential manner. The total number of sensors used in this configuration was 58. For dataset3, five strips were located over extensor and flexor muscle bellies and on the anterior side of the forearm. The total number of sensors used in this configuration was 37. More detail on dynamic datasets can be found in Ahmadizadeh et al. (2017).

#### 2.2.2. Protocol

**Figure 3:**
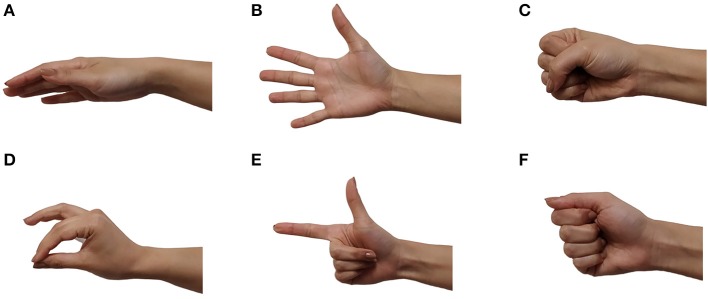
The six grips used in this protocol: **(A)** relax, **(B)** open, **(C)** force, **(D)** tripod, **(E)** finger point, **(F)** key.

The protocol used in this study consisted of six grips: relax, open, force, tripod, finger point, and key as shown in Figure 3. This set of grips was chosen to include neutral hand gestures and also functional movements important in activities of daily living (ADL) (Cho et al., 2016; Ahmadizadeh et al., 2017; Ferigo et al., 2017).

**Figure 4:**
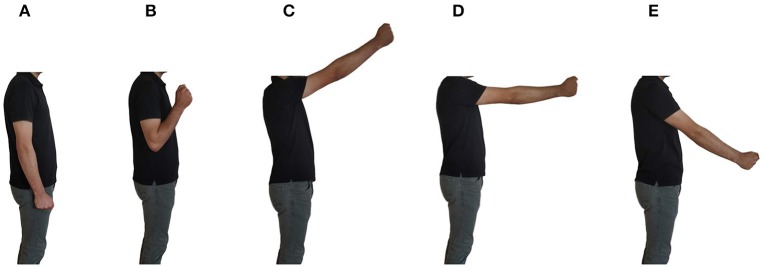
The dynamic protocol used for dataset2 and dataset3. Participant moved his arm through positions **(A–E)** in a circular motion.

To collect data, the subject was asked to wear the custom designed prosthesis. No preparation was needed prior to donning the prosthesis. He would then perform the six grips of the protocol and hold them for 15–25 s. This process was done for five repetitions with rest in between as needed. For dataset1, gestures were performed as the subject held their arm in a stationary position with their elbow flexed at 90°. For dataset2 and dataset3, subject held the grips as he was moving his arm in the circular dynamic motion shown in Figure 4. FSR data were collected at sampling rate of 10 Hz (Ferigo et al., 2017; Jiang et al., 2017).

### 2.3. Data Analysis

In this section, feature selection (FS) algorithms used in this study are explained. It is also explained how the data were analyzed and what outcome measures were used to compare selected FS algorithms.

In this study, no feature extraction was performed and each feature (channel) corresponded to one of the sensors. As a result, feature selection (channel selection) selects sensors whose values had the most contribution to the gesture classification accuracy. At each sample point (10 samples per second), input to the classifier was the combination of sensor values at that point in time from all selected sensors (features). There were 10 classification outputs per second each corresponding to one of the sample points as the sampling frequency is 10 Hz.

#### 2.3.1. Feature Selection Algorithms

As explained in the background section, feature selection algorithms can be categorized to three major classes: filter methods, wrapper methods, and embedded methods (Pal and Foody, 2010; Xu et al., 2010; Yan and Zhang, 2015). Wrapper methods are then split to two main categories depending on their method of selecting features in each of their iterations: sequential selection algorithms and Heuristic algorithms (Chandrashekar and Sahin, 2014).

Here, one commonly used algorithm from each of the aforementioned categories was chosen and its performance was assessed when applied to data collected from the pilot subject. Selected algorithms for this study were: minimum redundancy, maximum relevance (mRMR) which is a filter method (Hanchuan et al., 2005; Chandrashekar and Sahin, 2014), Sequential forward selection (SFS) (Chandrashekar and Sahin, 2014) as the sequential selection wrapper method, Genetic algorithm which is a heuristic wrapper method (Liu et al., 2002; Krishnaswamy et al., 2011; Chandrashekar and Sahin, 2014), and Random forest as the embedded method (Pal and Foody, 2010; Duro et al., 2012).

MRMR is an FS algorithm that improves on mutual information and attempts to choose a feature subset that minimizes redundancy defined as having highly correlated features in the chosen subset. It also tries to maximize relevancy so that chosen features are highly informative about their corresponding class labels (Ding and Peng, 2005).

The implementation of mRMR used in this study maximized relevancy of selected features with the targeted class, which is represented by the mutual information of the feature and the targeted class. It also tries to minimize redundancy of the selected features with each other which is quantified using the mutual information of each feature with all other features in the feature subset. The quantity that is maximized in this algorithm is the following (Ding and Peng, 2005):

(1) $$\begin{array}{r} \frac{I\left(a,l\right)}{\frac{1}{\left|F\right|}\sum_{b\in F}I\left(a,b\right)} \end{array}$$

Where *l* is the targeted class, *a* is the feature being investigated, *I* is the mutual information of two variables, *F* is the subset of features, and |*F*| is the number of features in the subset.

A third-party implementation of mRMR for MATLAB was used to apply the Mutual Information Quotient criterion (MIQ) version of the algorithm on collected data. MIQ was chosen since it has been recommended for discrete features in a study by Ding et al. For more details on the implementation of the algorithm, refer to Ding and Peng (2005).

The selected sequential wrapper algorithm for this study, SFS, selects features by starting with an empty set and adding one feature in each iteration. The feature added in each step is chosen so that the objective function, which is defined as the LOOCV classification, is minimized. This can be represented by the following steps (Li et al., 2017):

(2) $$\begin{array}{l} \begin{array}{c} SF_{0}=\emptyset \\ RF_{0}=all\;features\\ For\;i\;=\;1\;to\;n\\ \;accuracy\left(SF_{i}\right)=\underset{b\in RF_{i-1}}{Max}\left(accuracy\left(SF_{i-1}\;+b\right)\right)\\ \;RF_{i}=RF_{i-1}-a\\ \;SF_{i}=SF_{i-1}+a\\ end \end{array} \end{array}$$

Where *SF*_*i*_ is the selected feature subset in iteration *i*, *RF*_*i*_ is the remaining feature subset (not yet selected) in iteration *i*, and *n* is the number of all features

MATLAB's implementation of the algorithm was used with Linear Discriminant Analysis (LDA) as its classifier (Chandrashekar and Sahin, 2014). Information about settings used for the two stopping criteria used for this algorithm is provided in the next section.

Genetic algorithm is an evolutionary optimization algorithm (Chandrashekar and Sahin, 2014) that is used in this study as the Heuristic FS method. In this application,GA tries to optimize (minimize in our case) the objective function which was defined as the LOOCV classification error using LDA classifier in this experiment. The algorithm's MATLAB implementation was used for this purpose. Due to inherent randomness of GA, the process was performed 10 times for each dataset and for each outcome measure, and average values were reported. GA was implemented using uniform mutation, tournament selection, arithmetic crossover, elite cont of 2, and random initial population. Information about stopping criteria used for GA is provided in the next section.

For the embedded feature selection method, Boruta package for R was used. Boruta starts with creating shadow variables by copying original variables and shuffling their values. It then trains a classifier [random forest (RF)]) using the original and shadow variables. The importance score of original variables are determined based on comparison of their z-score with the z-score of shadow variables. This is performed in iterations until the final decision is made and feature scores are returned (Duro et al., 2012). In the implementation of Boruta, number of trees for RF was set to 500 and the maximum number of importance source runs was set to 100.

#### 2.3.2. Stopping Criterion

In order to decide what the optimum feature subset is, performance-based criterion was used. Performance base stopping criterion defines a condition based on system performance that determines when the algorithm stops and returns the resulting feature subset. Convergence conditions vary depending on the selection algorithm used.

One possible stopping criterion is the relative iterative improvement (RII) in classification accuracy. This method is suitable for SFS since the algorithm tries to improve classification accuracy in each iteration. In the implementation of SFS used in this study, the algorithm stopped when improvement in current iteration was less than a relative threshold defined by the following formula where *eps* = 2.2204*e* − 16 and *TolFun*, which determines the tolerance for termination based on improvement of the objective function (classification error in this case) value is set to 1*e* − 6:

(3) $$\begin{array}{r} critTh=oldCrit\left(abs\left(oldCrit\right)+sqrt\left(eps\right)\right)*TolFun \end{array}$$

In this formula, *critTh* is the threshold against which improvement of the objective function in each iteration is compared, and *oldCrit* is the value of the objective function in the previous iteration.

Another stopping criterion used in this study was the global maximum. It is used for algorithms that output ranking of features or order features in term of their importance. Accuracy of feature sets with 1 to all features is calculated as features are added according to their rank and the feature subset resulting in the maximum accuracy is selected. In this study, global maximum was used for mRMR and Boruta. SFS was also examined using this criterion as well as RII.

The last stopping criterion used in this study was Function Tolerance over Stall Generations, which is used for GA. This is also a performance criterion that concludes the process when average performance enhancement over a predefined number of generations is less than a threshold. In the analysis done for this study, function tolerance was set to 1e-6 and maximum stall generations was set to 50.

To summarize, five feature selection methods were examined in this study: SFS with RII stopping criterion, SFS with global maximum stopping criterion, mRMR with global maximum stopping criterion, GA with function tolerance over stall generations stopping criterion, and Boruta with global maximum stopping criterion.

#### 2.3.3. Algorithm Assessment

Chosen feature selection algorithms were assessed based on three outcome measures: their running time, the algorithms' performance in terms of classification accuracy, and their stability.

Reported running time for each of the methods for each dataset is the amount of time it took for the implementation of the method used in this study to run. Running time is an indication of the algorithm's computational complexity and can be important in cases where computational power is limited or the sample sizes are large.

##### Classification accuracy

The goal of using FS in this study was to reduce input dimension with no significant loss of vital information regarding gesture classification. As a result, classification accuracy is the first outcome method used for assessment of the five FS methods.

**Figure 5:**
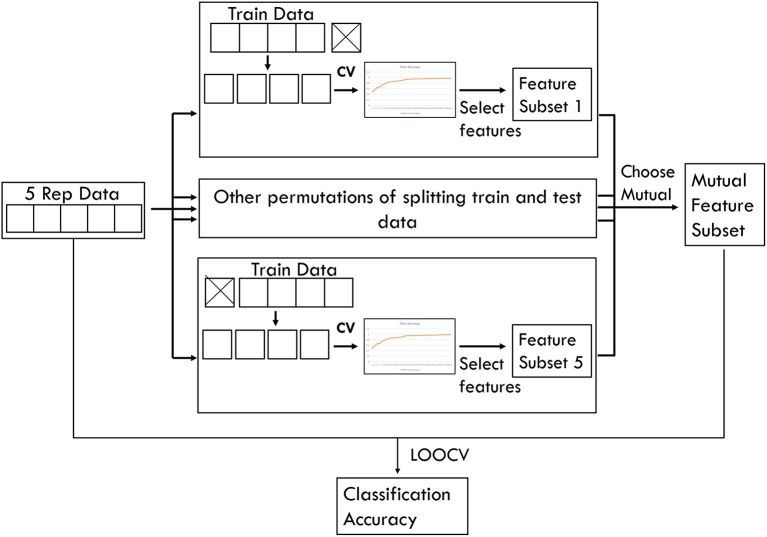
Feature selection process used in this study.

To obtain classification accuracy yielded by each feature selection algorithm in this study, leave-one-out cross validation (LOOCV) was used to separate one repetition of collected data as test data and the remaining four repetitions as training data. Feature subset was chosen using LOOCV on the four repetitions of training data. This process was repeated for the five permutations of test data. Then only the features that were mutually selected in all five iterations were chosen for the final feature subset. This process is illustrated in Figure 5. Reported classification accuracies were the result of performing LOOCV on all five repetitions of data using only the selected features. These accuracies were compared with the baseline accuracy.

##### Stability

As Defined by Chandrashekar et al., stability of a feature selection method is consistency of the feature subset chosen by the algorithm as new training data are introduced or when some training data are removed (Chandrashekar and Sahin, 2014). Stability is an important outcome measure for comparison of algorithms used in this study since it determines the algorithm's sensitivity to the size of training data. Feature subset variations observed in this study could also be affected by variability of data in different repetitions. Variability of data in applications for individuals with amputation is inevitable due to limited visual feedback. This makes stability an important measure for applications similar to the one examined here.

To compare stability of different algorithms selected for this study, selected feature subsets were compared when training data were reduced from the first four repetitions to the first three repetitions and then to the first two repetitions. For all these cases, the last repetition was used as test data. For this outcome measure, variation in feature subsets were compared using an average percentage of variation in each iteration as training data were reduced, which was obtained using the following formula:

(4) $$\begin{array}{lll} Variation\% & = & \left(number\;of\;features-number\;of\;mutual\;features\right)\\ \; & \; & /number\;of\;features*100 \end{array}$$

## 3. Results

In this section, results for assessment of the five feature selection methods are reported and compared with the baseline accuracies. Baseline accuracies for the six gestures used in this study were 86.45 ± 4.5%, 70.7 ± 10.6%, and 80.4 ± 11.4% for dataset1, dataset2, and dataset3, respectively.

### 3.1. Classification Accuracy

**Figure 6:**
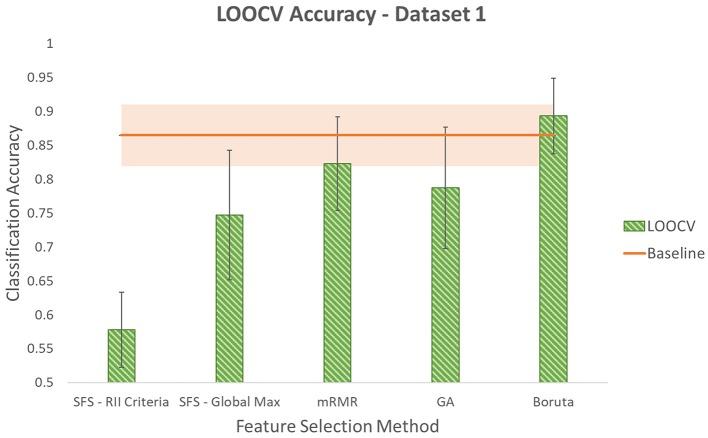
Leave-One-Out Cross Validation accuracy using different feature selection methods for dataset1. Shaded area around the baseline indicates its standard deviation.

**Figure 7:**
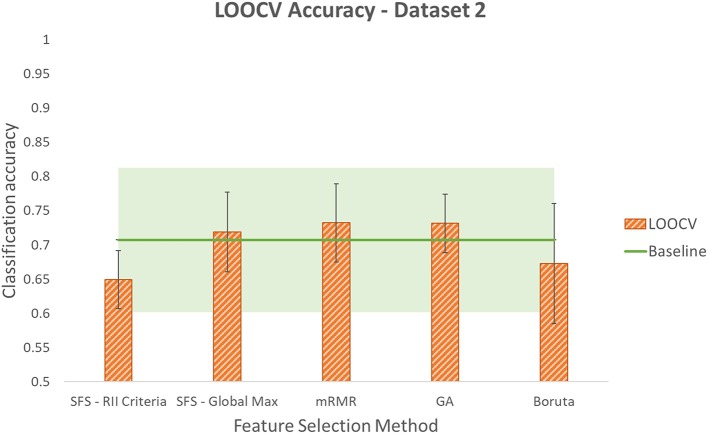
Leave-One-Out Cross Validation accuracy using different feature selection methods for dataset2. Shaded area around the baseline indicates its standard deviation.

**Figure 8:**
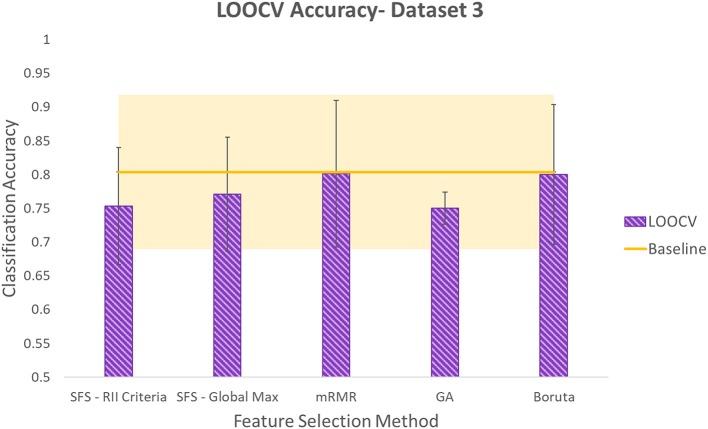
Leave-One-Out Cross Validation accuracy using different feature selection methods for dataset3. Shaded area around the baseline indicates its standard deviation.

Accuracies obtained using LOOCV with selected features by each of the five methods for the three datasets are shown in Figures 6–8. Each method produced a feature subset with a different number of features which are shown in Table 1.

**Table 1:** Number of features selected in LOOCV using different feature selection methods.

	**SFS-RII criteria**	**SFS-global max**.	**mRMR**	**GA**	**Boruta**
Dataset1	2	12	23	11.6 ± 1.3	27
Dataset2	8	14	35	16 ± 1.8	32
Dataset3	11	15	30	11.8 ± 1.0	29

GA was run 10 times and reported values in Table 1 indicate the average of the 10 times. The lowest numbers of features selected by GA were 10, 14, and 10 for dataset1, dataset2, and dataset3, respectively. These feature subsets resulted in classification accuracies of 78.4, 71.5, and 71.4%, respectively.

To determine whether reduction in the number of input channels caused a significant decrease in classification accuracy, the Student's paired *t*-test at significance level of 5% was used. It is worth noting that this is an exploratory study with one participant and five repetitions of the protocol for each dataset. Each reported classification accuracy is the average of five permutations of LOOCV. Each of these five values are the average classification accuracy of six gestures that are performed in each repetition. The values used in the *t*-test are the five LOOCV accuracies. Given the low sample size of the *t*-test, low power is expected and results should be considered with caution. This is one of the limitations of this study and it would be valuable to do an experiment with a larger sample size in future work. Even though the power of the *t*-test is low, looking at the average and standard deviation values, it seems that in populations that do not prove to be significantly different using *t*-test, the difference between averages is not considerable considering standard deviations.

It was found that using SFS with either of the two stopping criteria resulted in accuracies that were significantly lower than the baseline in dataset1. However, in this dataset, no significant difference was determined for the other three methods: mRMR, GA, and Boruta. In dataset2, no significant difference was observed comparing the baseline accuracy with the accuracies obtained using smaller feature subsets produced by any of the five feature selection methods. In dataset3, SFS with RII criteria lowered accuracy with significant difference. However, no significant difference was observed comparing accuracies produced by the other four methods and the baseline accuracy. It is worth noting that although GA resulted in a lower average accuracy compared to SFS-RII criteria, due to the small difference in the average accuracies of these two methods (0.31%) and GA's smaller standard deviation, the Student's *t*-test could not reject the null hypothesis that it comes from a population with the same mean as the baseline, while in the case of SFS-RII criteria, the null hypothesis was rejected.

Table 2 shows the grips with the lowest accuracy for each dataset using each FS method. In this table, the force grip appears frequently for dynamic datasets. A closer look at the errors of prediction for this class showed that in various cases, the force grip was misclassified as either the finger point grip or the key grip. This is likely because these grips are differentiated through fine finger movements. Variations in muscle volume caused by these movements could be affected by variations in the force exerted by the prosthetic socket as the user moves his arm dynamically.

**Table 2:** The grip with highest classification error for each dataset, using each FS method.

	**SFS-RII criteria**	**SFS-global max**.	**mRMR**	**GA**	**Boruta**
Dataset1	Finger point	Finger point	Force	Finger point	Tripod
Dataset2	Force	Force	Key	Force	Key
Dataset3	Force	Force	Force	Force	Force

Using SFS with either of the stopping criteria, grips with the lowest accuracies were “finger point” in dataset1 and force in dataset2 and dataset 3. Using mRMR, force was the grip with the lowest accuracy in dataset1 and dataset3, and key was the grip with lowest accuracy in dataset2.

To summarize, mRMR, GA, and Boruta were able to meet the goals defined in this study in all three datasets. Percentage decrease in number of features using each of the three methods are shown in Table 3.

**Table 3:** Percentage decrease in input features using different feature selection methods.

	**mRMR (%)**	**GA (%)**	**Boruta (%)**
Dataset1	63.5	81.6	57.1
Dataset2	39.7	72.4	44.8
Dataset3	18.9	68.1	21.6

### 3.2. Stability

**Figure 9:**
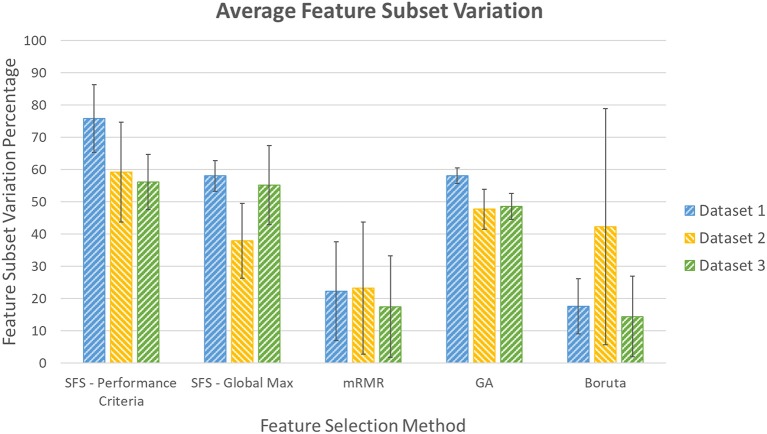
Percentage variation in feature subsets as amount of training data were decreased.

Stability of the five feature selection methods were measured based on consistency of the feature subset they select when number of samples in training data was decreased. This was done by comparing the variation percentage in feature subsets selected by different selection methods. These values for the three datasets are shown in Figure 9.

### 3.3. Running Time

The five FS methods considered in this study are different in terms of computational complexity and running time. Running times for the five methods for each dataset are shown in Table 4. MRMR and SFS-RII criteria have the shortest run time interchangeably for the three datasets. GA and then Boruta have the longest run times for all datasets.

**Table 4:** Running time for each FS method for each dataset (s).

	**SFS-RII criteria**	**SFS-global max**.	**mRMR**	**GA**	**Boruta**
Dataset1	90.5	311.1	54.7	2,592.3	1,504.5
Dataset2	71.8	133.5	107.2	1,721.2	163.5
Dataset3	42.1	53.3	42.5	2,112.1	111.4

## 4. Discussion

Baseline accuracies were comparable to that of other studies. Previous studies by Cho et al. and Ferigo et al. used the same 6 grips in a static protocol on four subjects and one subject with transradial amputations and reported classification accuracies of 62.61 ± 11.5% and 81.2 ± 11.3%, respectively. Ferigo et al. also used a dynamic protocol similar to the one used in dataset2 and dataset3 of this study and reported accuracy of 75.5 ± 9.2% (Cho et al., 2016; Ferigo et al., 2017). Other studies using EMG with subjects with amputation have reported classification accuracies in the range of 85–90% for 4–6 classes of movement (Peerdeman et al., 2011).

### 4.1. Classification Accuracy

This study focused on assessment of five feature selection methods on reduction of input dimension of an FMG controlled powered prosthesis without significantly sacrificing performance of the device. To determine if feature number reduction affected performance, classification accuracy was examined since it determines probability of correct prediction of user intentions.

Results showed that out of the five methods used to reduce input dimensions, while maintaining system performance, three methods were able to reduce the number of input features by 18.9–81% in the three datasets used in this study. This was possible since multiple sensors might produce correlated information depending on their location. Another reason is that some of the input channels could contain noisy or irrelevant signals and removing them could increase class separation and lead to higher classification accuracies (Guyon and Elisseeff, 2003; Kumar et al., 2005).

Elimination of irrelevant or noisy data through feature selection can also be observed by comparing standard deviations of the LOOCV accuracies over the five repetitions before and after feature selection, especially in dynamic datasets. Dynamic datasets are more prone to containing noisy or irrelevant data due to factors, such as weight of the prostheses applying compressive or tensile forces as the orientation of the arm changes, slight movements of the socket, and variations in movement of the arm. In the two dynamic datasets of this study, after feature selection, LOOCV standard deviations were decreased by 4.8 and 3.3% in dataset2 and dataset3, respectively, which indicates the possibility that eliminated features were contributing to increased data variation among various repetitions.

It is worth noting that even after feature selection, data variability is still evident through average LOOCV standard deviations of 7.3% in dataset1, 5.8% in dataset2, and 8.1% in dataset3 for the five FS methods. Standard deviations of about 11 and 9% were also reported by the two studies with datasets similar to the ones used here (Cho et al., 2016; Ferigo et al., 2017). It is likely that data variability in prostheses control applications is inevitable, especially as experimental setups become closer to the practical use case of the system. In future work, it would be valuable to conduct a study that differentiates between noisy or irrelevant signals and data variability that would be inevitable in real use case of the prostheses. This could potentially be achieved through studying variations among small sections of each repetition.

Comparing performance of the FS methods considered in this study, out of the three methods that could successfully achieve the goal defined in this study, GA resulted in the most reduction of input features in all datasets with a chosen feature subset of about 11 sensors out of the original 63 sensors resulting in average classification accuracy of 0.78 ± 0.09 in dataset1. It also reduced feature numbers to 16 sensors out of the total of 58 sensors in dataset2 yielding an accuracy of 73.2 ± 4.3% and a chosen feature number of about 12 sensors out of the total sensor number of 37 in dataset3 with resulting classification accuracy of 75.0 ± 2.4%.

Neither of the other two methods outperformed the other drastically in terms of feature number reduction in any of the three datasets. The reduction percentage difference for Boruta and mRMR in the three datasets were from 2.7 to 6.4 % while GA outperformed them by reducing feature numbers by up to 46.5% more in dataset3. This could be because wrapper methods, such as GA make their selection in each iteration based on classification accuracy. On the other hand, the other two methods (mRMR and Boruta) do not include classification in their selection decision. Thus, GA selects the feature subsets for the very specific application and tries to maximize the measure considered for it—i.e., classification accuracy. For this reason, if multiple classifiers or more generalized applications were to be considered, GA would not necessarily be able to produce similar results as the ones it produces when only one classifier is considered. Another drawback of wrapper methods, such as GA compared to Boruta and mRMR is their computation complexity. Wrapper methods perform a classification task in each iteration of the algorithm which could be computationally extensive in the presence of large sample sizes (Chandrashekar and Sahin, 2014).

Average accuracies that resulted from the three selected feature selection methods in dataset1 were within the range of “baseline accuracy—7.7%” and “baseline accuracy + 2.9%.” In this dataset, Boruta yielded the highest accuracy that was higher than the baseline accuracy. In dataset2, variation in accuracies produced by the three methods were less compared to dataset1. Contrary to dataset1, Boruta produced the feature subset with the lowest resulting accuracy compared to the other two. Accuracy obtained using the feature subset selected by Boruta in dataset2 was less than the baseline accuracy by 3.4% while the other two methods resulted in accuracies that were about 2.5% higher than the baseline accuracy. In dataset3, Boruta and mRMR both resulted in accuracies similar to the baseline accuracy while GA reduced the accuracy by 5.4%.

None of the three methods resulted in the highest classification accuracy in all three datasets. However, it is worth noting that a decrease of up to about 7% in accuracy when the average standard deviation was about 7.1% (in the accuracies obtained using the three selected methods in all datasets), which is not considerable especially in this case where no statistically significant difference was shown.

SFS with either of the stopping criteria was not able to meet the goal defined in this study. This could be since the algorithm, in iteration *i*, only considers selections combining the “selected feature subset (*SF*_*i*_)” and one of the feature in the “remaining feature subset (*RF*_*i*_)”—i.e., features that are not yet selected to be in the selected feature subset. This could be a drawback since there is a chance that a combination consisting of part of the features in *SF*_*i*_ and more than one feature from *RF*_*i*_ would produce better results.

To determine sensitivity of the proposed method to the size of datasets used for model training, an analysis was conducted using selected features by the five FS methods and only half of the data from each repetition. Results showed that reducing the size of the datasets to half their size would not significantly change classification accuracies in dataset1 and dataset3. Using only half of the data significantly increased accuracies obtained using feature subsets selected by SFS-Global Max. and GA methods in dataset2. This could be due to the eliminated data being noisy. Obtained classification accuracies using the smaller datasets are reported in Table 5.

**Table 5:** Classification accuracies obtained using half of the data.

	**SFS-RII criteria (%)**	**SFS-global max. (%)**	**mRMR (%)**	**GA (%)**	**Boruta (%)**
Dataset1	60.8 ± 7.5	76.7 ± 8.3	79.8 ± 5.1	75.8 ± 10.3	88.0 ± 5.0
Dataset2	66.5 ± 4.4	75.8 ± 4.5	74.6 ± 6.1	76.8 ± 3.5	71.3 ± 6.8
Dataset3	73.7 ± 9.1	77.6 ± 8.6	80.1 ± 10.0	75.8 ± 2.8	80.0 ± 8.6

Comparing this study with the studies by Wang et al. and Li et al. that performed channel selection for gesture classification (using sEMG), this study was conducted using both static and dynamic protocols with an individual with an amputation using a prosthetic socket. Experimental setup of this study was closer to the real use case of the application and was likely to be able to capture some of the possible errors introduced by the real application of the system. The aforementioned studies each investigated one FS method, while this study runs a comparison between five methods. In terms of feature number reduction, Wang et al. reported classification accuracy of 69.3% after about 35.7% decrease in channel numbers and Li et al. reported accuracy of 84.2% after about 79% decrease in the number of channels. In the study presented here, maximum percentage decrease in the number of channels varied between 57.1 and 81.6% in the three datasets (Li et al., 2017; Wang, 2019).

To summarize, all three methods resulted in inconsiderable reduction/increase in accuracy while GA resulted in the most reduction in feature number. And using only half of the data, comparable results were obtained.

### 4.2. Running Time

For the different datasets, either mRMR or SFS with RII criteria was the fastest method. After these, SFS-Global Max., Boruta, and GA had increasing running times in that order. MRMR was one of the fastest method since it does not perform any classification tasks and ranks features based on solving the value of the formula stated in Equation (1).

The running time for SFS with RII criteria method was less than the running time for SFS-Global Max. method. This is since the number of iterations for SFS-Global Max. is fixed and equal to the number of features as the algorithm starts with an empty set and adds one feature to it in each iteration until all features are added. On the other hand, the number of iterations of SFS-RII criteria is data dependent and is less than or equal to the number of all features.

After GA, Boruta is the method with the longest running time. Boruta's running time for dataset1 was longer compared to dataset2 and dataset3. This was because in analysis for dataset1, Boruta ran an average of above 90 runs to resolve all attributes left tentative for the five LOOCV cases, while for dataset2 and dataset3, all attributes were resolved in an average of about 13 runs. This is due to fluctuations of importance of some of the attributes requiring more runs for the algorithm to either confirm or reject them. There is also shuffling of values of shadow variables that is performed when shadow variables are created. Based on the result of this shuffling, running time of the algorithm could be affected. Although there are methods that fix convergence issues of the algorithm due to tentative attributes, these methods use weaker tests for attribute judgement. To summarize, the run time of Boruta is data dependent.

GA has the longest run time compared to all the other methods since it ran an average of about 84, 81, and 69 iterations for the five LOOCV cases of dataset1, dataset2, and dataset3, respectively. In each iteration, a classification task was performed in addition to steps of genetic algorithm. As mentioned before, in this study, feature selection using GA was performed 10 times for each dataset and each outcome measure. The run time reported in Table 4 is for one processing of the method for the “classification accuracy” outcome measure.

### 4.3. Stability

Stability of a feature selection method was the second outcome measure assessed in this study. It is worth noting that the variations shown in this measure did not affect the accuracies reported in the previous section since the accuracy results were obtained using only the features that appeared in all iterations of cross validation and features accounting for the variability were discarded.

As shown in the “Results” section, out of the three algorithms that were able to achieve the goals of this experiment, GA produced the feature subsets with most feature subset variation percentage ranging from 47.7 to 58.0% in the three datasets. This showed that GA was more sensitive to the volume of training data or variability in data that were collected in different repetitions of the experiment. This could be due to the randomness that is inherent to this algorithm. Comparing the other two methods, mRMR resulted in average feature subset variation of about 21% in the three datasets and Boruta resulted in average variation percentage of 24.8% in all datasets with more variability between the three datasets.

It is worth noting that in this experiment, as the size of training data was reduced, the total number of samples in training data was decreased by 25% in the first iteration, 33% in the second iteration, and 50% of the remaining data in the last iteration. As a result, the stability measure considered here represented sensitivity of methods to high variations in the size of training data. In applications where variability in the size of training data was not considerable, these methods might yield less instability, which is only due to sensitivity to variations of data in different repetitions.

## 5. Conclusion and Future Work

This study examined feasibility of using a high density FMG apparatus to determine optimum number and location of sensors for each individual. This aimed at design of a simpler and lower cost FMG controlled prosthesis with performance comparable to that of high density FMG systems.

Five feature selection methods were examined using data collected on a pilot subject with transradial amputation: SFS with two different stopping criteria, mRMR, GA, and Boruta. Three of the five FS methods were able to reduce the number of FSR sensors while maintaining vital information for gesture classification in all three datasets examined in this study.

Out of the three selected methods, none outperformed the others in all datasets in terms of classification accuracy, however, GA produced the smallest feature subsets in all datasets without significantly sacrificing performance defined by classification accuracy. MRMR and Boruta were more stable than GA in all datasets which means that GA was most sensitive to variations in training data.

This was a case study based on one individual and a specific prosthetic socket. This is because the socket was custom-made for the participant of this study and also due to the difficulty of recruiting individuals with amputations to participate in such studies. Potential future work includes expansion to more individuals and more classes of motion. This would make results more applicable to the general case of the application. Another focus of further investigations would be development of an easy-to-use high density FMG apparatus. Also, more feature selection methods could be assessed for a more thorough comparison of FS algorithms for this application.

Various studies have explored the use of different classifiers for applications similar to the one considered in this study. In most cases, using different classifiers did not result in significantly different results. For this reason and also since the focus of this study was channel selection, only one classifier (LDA) was used in this study. To build on findings of this study, it would be valuable to investigate the effect of using various classifiers on the datasets of this study. Collection of larger datasets from one or more individuals would introduce the possibility of using deep learning methods and LSTM which have shown promising results for gesture recognition in other studies.

Another limitation of this study was that it did not investigate the effect of noise reduction. This should be studies in future works. Also, the sample size used for the *t*-test was not enough to guarantee high test powers. In future work, a study should be conducted with more repetitions of data so that enough samples are available for high powers of *t*-test. Moreover, given that this was a pilot study, there is dependency of data and therefore the assumptions underlying the statistical method used are not fully met and results should be considered with caution.

Three datasets were used in this experiment to include variations in static or dynamic protocols, different sensor configurations, and different sample sizes. However, the reason why results obtained from these datasets varied was unaddressed. It would be valuable to investigates potential reasons for these variations in future work.

Location of selected sensors is a valuable information that is unaddressed in this study. Future work should include a study that explores if there are locations of the forearm that would be more optimal for placement of sensors in FMG controlled prostheses applications. For this purpose, the algorithm should be designed so that selection of sensors that are spatially close to each other are promoted.

It has been demonstrated that unlike EMG, performance of FMG does not rely heavily on feature extraction (Belyea et al., 2018) which has led to the common use of raw FMG signals for gesture recognition (Radmand et al., 2016; Ferigo et al., 2017; Jiang et al., 2017; Xiao and Menon, 2017a; Belyea et al., 2018). Since the focus of this study was channel selection, feature extraction was not considered in this experiment. It would be valuable to explore the feasibility of using feature extraction to enhance performance of the system in future work.

This study showed feasibility of the proposed design method to produce a system with performance comparable to that of the high density FMG systems with lower cost and less complexity.

## Data Availability Statement

The datasets generated for this study will not be made publicly available.

## Ethics Statement

The studies involving human participants were reviewed and approved by Office of Research Ethics at Simon Fraser University. The patients/participants provided their written informed consent to participate in this study.

## Author Contributions

CA developed the prototype used in this study and carried out the data collection in collaboration with BP. BP developed the custom prosthetic socket. CA performed the data analysis and wrote the content of this manuscript. CM supervised the research and provided guidance throughout engineering developments and data analysis.

### Conflict of Interest

BP was employed by company Barber Prosthetics Clinic. The Principal Investigator, CM, and members of his research team have a vested interest in commercializing the technology tested in this study, if it is proven to be successful and may benefit financially from its potential commercialization. The remaining author declares that the research was conducted in the absence of any commercial or financial relationships that could be construed as a potential conflict of interest.
